# Payment mechanism and medical quality: new evidence from China healthcare reform

**DOI:** 10.1186/s12913-026-14692-y

**Published:** 2026-06-08

**Authors:** Yaxuan Zuo, Yingtan Mu, Jiali Zhang, Qiuming Hu

**Affiliations:** The School of Public Administration, Southwestern University of Finance and Economics, 555, Liutai Avenue, Wenjiang District, Chengdu, Sichuan P. R. China

**Keywords:** DRG payment differential, Healthcare service quality, Incentive compatibility

## Abstract

**Background:**

Reform of payment methods promote to achieve the public value of health insurance through incentive-compatible mechanisms. Payment differentials, defined as the difference between DRG/DIP payment amounts and total medical expenses, together with the rational design of surplus retention mechanisms, are crucial for controlling healthcare costs, improving service quality, ensuring the sustainability of health insurance funds, and guiding medical institutions towards reasonable surplus targets.

**Methods:**

This study constructs a one-shot game model under conditions of information asymmetry among hospitals, patients, and health insurance agencies. Using sample data from health insurance settlements in DRG pilot cities for the years 2021–2022, with readmission rates as the quality indicator and payment differentials as the measurement index, the study explores the association of payment differentials on the service quality of medical institutions.

**Results:**

Negative payment differentials are significantly associated with higher readmission rates and lower measured service quality. This may be consistent with insufficient payment incentives under the DRG system, which prompts medical institutions to adopt more aggressive cost-control measures, such as reducing hospital stays and limiting expenditures on medical services, which in turn may be associated with lower service quality. Moreover, the relationship between payment differentials and service quality varies depending on factors such as the type of surgery, hospital tier, and patient age.

**Conclusions:**

There should be a more effective alignment between medical service pricing and health insurance payment standards, may help guide medical institutions toward more sustainable financial performance through the optimization of treatment processes, and promoting the shift towards a value-based payment system, potentially contributing to improved incentive alignment. Noting that as an observational study, our findings reflect correlations rather than causal effects.

## Background

Payment method reforms aim to achieve the public value of health insurance by introducing incentive-compatible mechanisms, guiding healthcare providers to balance their pursuit of self-interest with the goals of ensuring the sustainability of health insurance funds and improving patient health outcomes [1–3]. Existing studies have found that the Diagnosis-Related Groups (DRG) payment system generates positive incentive effects, effectively curbing unreasonable increases in medical costs, enhancing transparency and flexibility in healthcare delivery, and reducing hospital length of stay [4, 5]. However, other research highlights potential negative incentives, including limited cost-control effects, insufficient service provision, and quality deterioration associated with the DRG payment model [6–8].

A key metric in evaluating DRG/DIP payment systems is the payment differentials—defined as the difference between DRG/DIP payment amounts and total medical expenses^1^. This metric reflects the financial status of healthcare institutions under these payment models: a positive payment differentials indicates surplus retention for the health insurance fund, while a negative gap suggests financial loss. Reasonable payment differentials are fundamental to effective cost control and improved healthcare quality. However, excessively small payment differentials may incentivize providers to engage in undesirable practices such as upcoding, “cherry-picking”, shortening hospital stays, or reducing necessary medical services, potentially compromising care quality and harming patient health [9, 10]. Therefore, the crux of the issue lies in scientifically designing payment differentials and surplus retention mechanisms to guide healthcare institutions toward reasonable cost control and achieve incentive compatibility.

Therefore, this study employs sample data from health insurance settlement records in DRG pilot cities to examine the association between payment differentials and healthcare service quality, using readmission rates as a quality indicator and payment differentials as a measurement metric. The analysis is structured around the following aspects: the effect of payment differentials on patient readmission rates and the underlying mechanisms; and whether the impact of payment differentials on readmission rates varies by surgical type, hospital level, or patient age.

### A review of DRG payment’s effect on healthcare quality indicators

The impact of the DRG payment system on healthcare service quality has been a focal point in both domestic and international research. Previous studies have primarily analyzed indicators such as mortality and readmission rates, but no consensus has been reached on the findings [11].

The first perspective suggests that the DRG payment system may lead to a decline in healthcare service quality [12, 13]. The reasons include: (1) Healthcare providers may reduce service quality or limit necessary treatment measures, resulting in underprovision of services. For instance, Mayer-Oakes SA et al. (1988) found a 31% reduction in the number of intensive care unit beds under the DRG system [14]. (2) Hospitals may adopt practices like splitting admissions, which can degrade treatment outcomes and increase unplanned readmission rates. Kosecoff J et al. (1990) observed a 5% rise in unstable discharges [15], while Rogers WH et al. (1990) reported longer rehabilitation times in nursing facilities for patients discharged under the DRG system [4]. (3) Hospitals, constrained by cost considerations, may reduce investment in new medical technologies, limiting the adoption of innovative therapies and patients’ treatment options, thereby affecting service quality [16].

The second perspective argues that the DRG payment system does not necessarily increase readmission rates or degrade service quality, as effective integration with home healthcare and outpatient services may mitigate these effects [17].

The third perspective contends that DRG-based payment reforms incentivize providers to enhance efficiency and standardize practices, thereby improving healthcare quality [18, 19]. For example, Jian WY et al. (2015) evaluated the outcomes of Beijing’s 2012 DRG pilot program, finding reduced hospital stays, increased two-week readmission rates, and higher case mix index values [20]. Overall, the DRG system can optimize service structure, enhance efficiency, and balance interests among stakeholders by reducing cases, lowering case weights, and improving patient outcomes and healthcare worker value. However, if DRG reforms fail to incentivize providers to optimize services, patients may face quality issues, leading to adverse outcomes for multiple stakeholders [8].

Currently, research on the impact of insufficient payment incentives on healthcare quality remains limited. One perspective posits that insufficient incentives reduce quality [21]. For example, Fässler M et al. (2015) found that insufficient incentives reduced provider income, dampened motivation, and compelled cost-cutting measures such as service reductions or quality compromises [22]. Additionally, insufficient incentives may curtail investment in equipment upgrades, technology acquisition, and staff training [23]. Conversely, another perspective suggests minimal or negligible negative effects. For instance, Cots F et al. (2011) reported that while insufficient incentives under the DRG model may reduce certain necessary services, core service quality and key health indicators generally remain stable [24].

In summary, existing research has explored the impact of the DRG system on healthcare quality, focusing on mortality, readmission rates, cost control, and treatment outcomes, using diverse methodologies and perspectives. However, while some studies have examined the role of incentive mechanisms, there remains a lack of detailed analysis of how insufficient incentives influence provider behavior and its association with healthcare quality.

This study addresses these differentials by sample data from health insurance settlements in representative cities to investigate the effects of payment differentials under the DRG system on healthcare quality. The marginal contributions of this study are as follows: First, unlike previous research focusing on payment system reforms broadly, this study emphasizes the optimization and refinement of specific payment rules. Second, by analyzing the mechanisms through which payment differentials affect patient readmission rates, this research expands the perspective on the relationship between economic efficiency and service quality under the DRG model, providing new empirical evidence for policy evaluation. Third, the study further elucidates the incentive effects of the DRG system, examining how payment differentials influence provider behavior and the pathways of quality changes. Finally, using real-world sample data from health insurance settlements, this study conducts an empirical analysis of the actual effects of DRG payment policies in representative Chinese cities, offering valuable academic insights for advancing policy design in DRG reform.

## Methods

### Model framework

Diseases are categorized into mild and severe, with probabilities *x* and *1 - x* respectively. Correspondingly, there are two treatment options: simple treatment with a cost of ${c_1}$ and complex treatment with a cost of ${c_2}$, $0 \leq {c_1} \leq {c_2}$. Simple treatment can cure mild diseases but is ineffective for severe cases, while complex treatment cures severe diseases and can also treat mild cases but yields a surplus.

The patient’s utility function is $U(H,Y){\mathrm{=}}H \times Y$ [25–31],^2^ with $U^{\prime}(\cdot)>0$ and $U^{\prime\prime}(\cdot)<0$. Here, H represents the treatment outcome (*H=1* for cured and *H=u* for uncured, *0 ≤ u<1*), and *Y=y - p* denotes the patient’s net income. The disposable income is y, and p is the insurance reimbursement rate, which is private information represented by the distribution function $\Psi (\cdot)$.

The patient’s co-payment ratio is $\theta \in (0, 1)$, assumed to be an exogenous variable.

The health insurance agency implements the DRG payment system, with monopsonistic power to set a uniform payment standard p for all treatments within the same diagnosis group. The agency’s objective is to minimize expenditures while maximizing patient health outcomes, represented as *V(p,H,x,θ,U)*, where C is total expenditure, H is overall health, and $\frac{{\partial V}}{{\partial p}}<0$, $\frac{{\partial V}}{{\partial H}}>0$.

### Constraints

When a patient receives treatment from a medical institution, the utility is *U(y - θ p)*; if the patient does not receive treatment from a medical institution, the utility is *U(uy)*. In order to ensure that the utility comparison of treatment is higher than that of not receiving treatment $U(y - \theta p) \geq U(uy)$, it can be concluded $y \geq \frac{{\theta p}}{{1 - u}}$ based on $U^{\prime}(\cdot)>0$. For a medical institution, the probability of a patient receiving treatment is $1 - [\Psi (y \geq \frac{{\theta p}}{{1 - u}})]$, and the corresponding profit is $\{ 1 - [\Psi (y \geq \frac{{\theta p}}{{1 - u}})]\} [p - c]$, which represents the cost required for treatment.

Define the expected profit function of the medical institution in the current period $\pi (p,c)=\{ 1 - [\Psi (y \geq \frac{{\theta p}}{{1 - u}})]\} [p - c]$.

The condition for a medical institution to accept medical insurance pricing is that the income of the medical institution is greater than 0, that is,

$$(1 - x)\pi (p,{c_1})+x\pi (p,{c_2}) \geq 0\:\:\:\left(\mathrm{IR}\right)$$

Unreasonable charges refer to the cost of complex treatment for patients with mild illnesses, and the medical institution deliberately diagnoses them as severe illnesses and charges for complex treatments, but actually implements simple treatments; second, when a patient has a mild illness, the medical institution deliberately diagnoses them as severe illnesses and actually performs complex treatments. The conditions under which medical institutions will not make unreasonable charges are:

When the illness is mild $\pi (p,{c_1}) \geq \pi (p,{c_2})$; When the illness is severe $\pi (p,{c_1}) \leq \pi (p,{c_2})$.

Insufficient diagnosis and treatment refers to the patient having a severe illness, and the medical institution can deliberately diagnose it as a mild illness and only implement simple treatments. In this way, it may make a profit in the current period, but will lose trust from the next period, and repeat the static equilibrium results from then on, and the subsequent profit of the medical institution is 0. The condition that medical institutions will not provide insufficient treatment is:

$$\begin{aligned}&\pi (p,{c_2})+\frac{\delta }{{1 - \delta }}[(1 - x)\pi (p,{c_1})\cr&\quad+x\pi (p,{c_2})] \geq \pi (p,{c_1})\end{aligned}$$

The left side of the above formula is the discounted total benefit when the medical institution provides honest treatment when the patient is seriously ill, and the right side is the discounted total benefit when the medical institution provides insufficient treatment. After simplification, we get:

$$\pi (p,{c_2})(1 - \delta +x\delta) \geq \pi (p,{c_1})(1 - 2\delta +\delta x)$$  

When the incentive compatibility conditions *V(p,H)* and participation conditions related to the medical institution are met, there is no deception on the equilibrium path, and the medical insurance institution will get *V(p,1)* in each period. The medical insurance institution also has another choice $p={c_1}$, and the utility obtained is $V({c_1},1 - x+xu)$. If the medical insurance institution is willing to encourage the medical institution to adopt an honest strategy, then there must be $V(p,1) \geq V({c_1},1 - x+xu)$, ${c_1} \leq p \leq {c_2}$. In summary, the optimization problem of the medical insurance institution is:

$$\mathop {\hbox{max} }\limits_{p} V(p,1)$$

$$\pi (p,{c_1}) \geq \pi (p,{c_2})$$

$$\pi (p,{c_2})(1 - \delta +x\delta) \geq \pi (p,{c_1})(1 - 2\delta +\delta x)$$  

$$V(p,1) \geq V({c_1},1 - x+xu)$$

### Optimal solution

Because *π (p,c)=*$$\left\{ 1 -\right.$$$$[\Psi (y \geq$$$$\left.\frac{{\theta p}}{{1 - u}})]\right\}$$*[p - c]*, so *p ≥*$$x{c_2}+$$*(1 -*$$x){c_1}+$$$$\frac{{1 - \delta }}{\delta }$$$$({c_2} - {c_1})$$. And because $$\frac{{\partial V}}{{\partial p}}$$*<0*, so the optimal solution is $${p^*}=$$$$x{c_2}+$$*(1 -*$$x){c_1}+$$$$\frac{{1 - \delta }}{\delta }$$$$({c_2} - {c_1})$$. In order for the medical institution to maintain its reputation (adopt an honest strategy), the medical insurance institution cannot lower the price to equal the average cost $$x{c_2}+(1 - x){c_1}$$; it should allow the medical institution to obtain a certain profit, that is $$\frac{{1 - \delta }}{\delta }({c_2} - {c_1})$$, there is a “reputation premium”.

The difference p-c (payment differentials) directly affects providers’ economic incentives. When p − c < 0, financial pressures may compel providers to adopt excessive cost-saving measures, such as reducing services or shortening hospital stays, potentially harming patient health outcomes and service quality.

#### H1

There is a significant negative relationship between the payment differentials and patient healthcare quality.

The payment differentials also influences treatment choices and care processes. Narrow profit margins may lead providers to adopt conservative or cost-controlling strategies, resulting in inadequate treatment for severe cases and increased readmissions.

#### H2

The payment differentials affects patient healthcare quality through provider behaviors.

The impact of the payment differentials on provider behaviors varies across contexts, such as hospital level, treatment type, and patient characteristics. High-level hospitals with greater resources may offset narrow payment differentials through external funding, maintaining care quality. Conversely, resource-constrained hospitals may exhibit reduced service quality and increased readmissions. Similarly, complex treatments requiring higher costs are more sensitive to payment differentials. Patient age also influences the extent of this impact, as older patients typically require more resources.

#### H3

The effect of DRG payment differentials on patient healthcare quality exhibits heterogeneity, moderated by treatment type, hospital level, and patient age.

### Data description

This study utilizes health insurance settlement sample data from DRG pilot cities for the years 2021–2022. The dataset includes information on individual patients, diagnoses, treatment details, healthcare institution characteristics, and costs derived from institutional health insurance settlement data and hospital admission records.

Individual Information: Includes unique patient identifiers, gender, age, and other demographic details.

Diagnosis and Treatment Information: Covers disease diagnosis names, ICD-10 codes, admission and discharge dates, DRG codes, length of hospital stay, number of surgical procedures, and more.

Healthcare Institution Information: Provides details such as institution name and hospital level.

Cost Information: We employ total hospitalization costs as our empirical measure of total medical expenses, calculated from health insurance settlement data and hospital admission records. This metric encompasses all direct medical cost categories incurred during hospitalization, including: (1) general medical service fees (registration, consultation, bed, and nursing fees); (2) diagnostic and examination fees (laboratory tests, imaging studies including CT, MRI, and X-ray, and pathology examinations); (3) therapeutic procedure fees (surgical fees, anesthesia fees, and treatment fees); (4) drug costs (western medicine, Chinese patent medicine, and herbal medicine); and (5) consumable costs (surgical supplies, interventional materials, and disposable medical items). From an economic perspective, total hospitalization costs represent the overall expense of inpatient services, equal to the sum of insurance reimbursements and patient out-of-pocket payments. This indicator reflects the direct medical burden on patients and the actual resource consumption of inpatient care.

However, several caveats should be noted. First, this measure captures only direct medical costs and excludes indirect operational costs such as hospital administrative expenses, fixed asset depreciation, capital investments, and personnel training costs. Consequently, total hospitalization costs underestimate the full economic cost incurred by healthcare providers. Second, under drug zero-markup and centralized procurement policies, drug and consumable prices approximate procurement costs; however, medical service prices remain subject to government price controls and may deviate from true service costs. Third, regional and institutional variations in pricing standards may affect cost comparability across different hospitals. Fourth, potential behavioral responses by providers (e.g., increased diagnostic testing to compensate for underpriced services) may cause measured expenses to deviate from “necessary” resource consumption. Despite these limitations, total hospitalization costs remain the most direct and accessible indicator for measuring patients’ disease economic burden and actual insurance protection levels, aligning with this study’s focus on the patient perspective of medical expense burden.

To capture variations in provider revenue, this study uses *łn c*, the natural logarithm of the ratio between the medical expenses reimbursed under the DRG payment system and total medical expenses. Here, r_c represents the institutional revenue adjustment under DRG-based payment compared to project-based payment.

Table 1 presents the descriptive statistics of the sample. Key observations include: The 30-day readmission rate is 17.5%, and the 60-day readmission rate is 20.7%, indicating relatively high readmission rates. The average patient age is 70.20 years, with a range spanning from 19 to 110 years. The age distribution is diverse, as reflected by a standard deviation of 16.18, predominantly comprising elderly patients. Hospital-level distribution is primarily concentrated in secondary and tertiary institutions. Regarding medical services, 55.5% of patients underwent surgical procedures. The average number of additional diagnoses per patient is 6.038, ranging from 0 to 16, highlighting the complexity of medical conditions in the sample population.

**Table 1:** Descriptive statistics (*N* = 115544)

Variable	Mean / Proportion	Variance	Minimum	Maximum
lnc	0.778	0.306	0	5.39
Whether to be readmitted within 30 days	0.175	0.380	0	1
Whether to be readmitted within 60 days	0.207	0.405	0	1
Male	58.9%	0.492	-	-
Age	70.20	16.18	19	110
Hospital grade	2.669	0.601	1	3
Whether to have surgery	0.555	0.497	0	1
Number of other diagnoses	6.038	4.425	0	16
Length of stay	11.39	10.99	0	65
Number of other diagnoses	12,294	10,522	1298	48,268
the institutional revenue adjustment (r_c)	-0.0640	0.663	-3.7	0.8

Data source: The author sorted out the data based on the sample data of medical insurance settlement

### Model design

The relationship between the payment differentials and healthcare quality provides a crucial lens for understanding the incentive mechanisms within health insurance systems. Under the DRG payment model, payment discrepancies directly influence the quantity and quality of services provided by healthcare institutions. This relationship reflects how quickly and to what extent providers adjust their service quality in response to payment differentials.

**Fig. 1:**
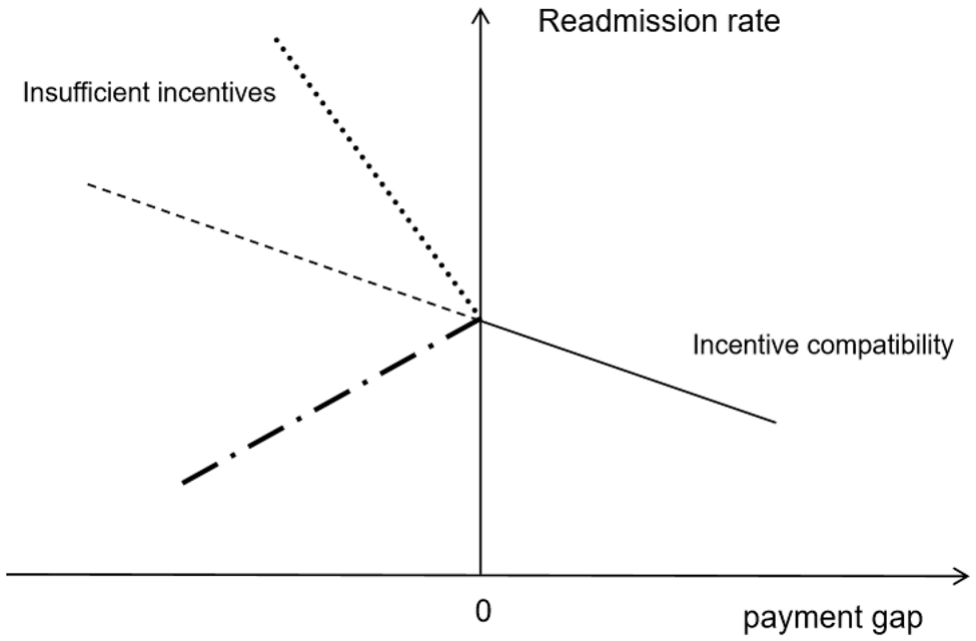
Incentive compatibility vs. Insufficient incentives

Specifically, when faced with a negative payment differentials (i.e., insufficient incentives), institutions often make rapid adjustments to mitigate financial pressure, such as reducing service quality. This leads to poorer patient outcomes and increased readmission rates, demonstrating the sensitivity of institutions to insufficient incentives. Conversely, a positive payment differentials provides additional revenue, incentivizing quality improvements. However, due to the complexity and gradual nature of quality enhancement processes, institutional responses to positive incentives tend to lag, limiting the speed of reductions in readmission rates (see Fig. 1).

Understanding this relationship is critical for optimizing payment policies and designing incentive mechanisms that balance service quality and cost control. To analyze this, a logistic regression (LOGIT) model is constructed:

$$\begin{aligned}\ln (\frac{p}{{1 - p}})&={\beta _0}+{\beta _1}\ln c+{\beta _2}\ln c \cdot D\cr&\quad+{\sum\limits_{i} {{\beta _i}{Z_i}} _{}}+\sum\limits_{i} {yea{r_i}} \cr&\quad+\sum\limits_{i} {diseasegrou{p_i}} +{\mu _{}}\end{aligned}$$

*P* represents probability of patient readmission within 30 days after discharge. *łn c* represents logarithm of the ratio of revenue under the DRG payment system to total medical expenses, capturing institutional revenue variation. D represents dummy variable representing the direction of the payment differentials. D = 1 if DRG revenue is less than total medical expenses (negative gap), and D = 0 otherwise. Z represents control variables, including gender, age, hospital level, surgical status, and the number of other diagnostic codes. Year and diseasegroup represent year fixed effects and disease group fixed effects, respectively. The regression coefficients ${\beta _0}{\beta _1}{\beta _2}$ capture the effects of revenue changes on readmission rates: When D = 0(positive payment differentials), ${\beta _1}$ represents the percentage increase in readmission rates for each percentage point increase in surplus. When D = 1 (negative payment differentials), the combined coefficients represent the percentage decrease in readmission rates for each percentage point increase in loss. If ${\beta _2}<0$ and statistically significant, this indicates that the rate of decline in readmission rates during revenue loss is slower than the rate of increase during revenue surplus, highlighting the asymmetric impact of payment differentials on healthcare quality (see Fig. 1).

## Results

### Descriptive statistical analysis

#### Changes in payment differentials averages

**Fig. 2:**
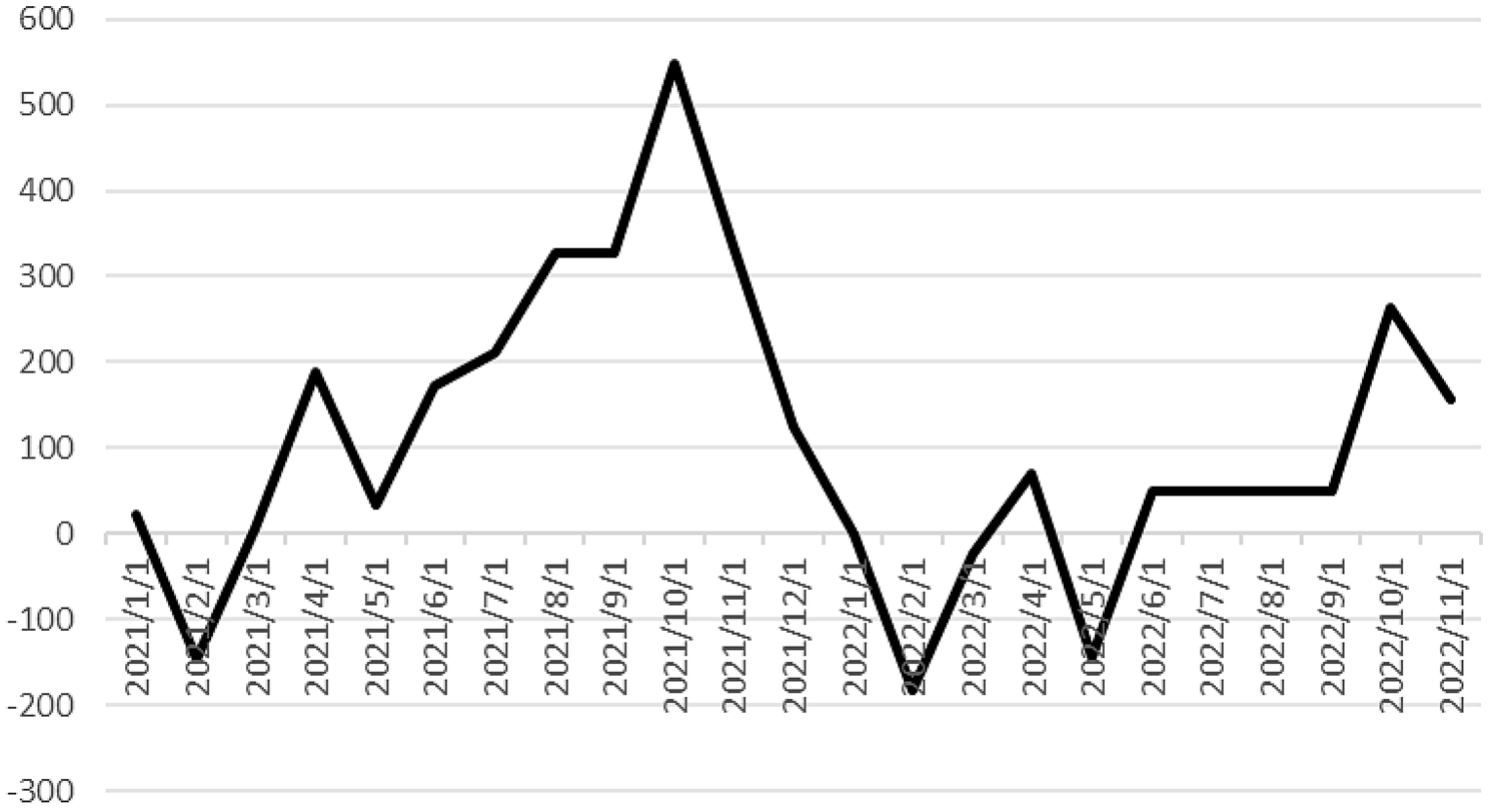
Time series of total payment differentials. Data Source: Compiled by the author based on health insurance settlement sample data

The total payment differentials was positive and showed an increasing trend in the early stages of policy implementation, indicating initial cost-containment effects and increased payment differentials for healthcare providers (Fig. 2). Over time, the total payment differentials stabilized at a slightly surplus level. This temporal pattern may reflect potential behavioral adjustments by providers in response to the DRG payment reform, as well as improvements in cost control and operational efficiency. It may also be related to increased competition as DRG payment levels gradually approached market averages [32, 33].

#### Changes in healthcare quality

**Fig. 3:**
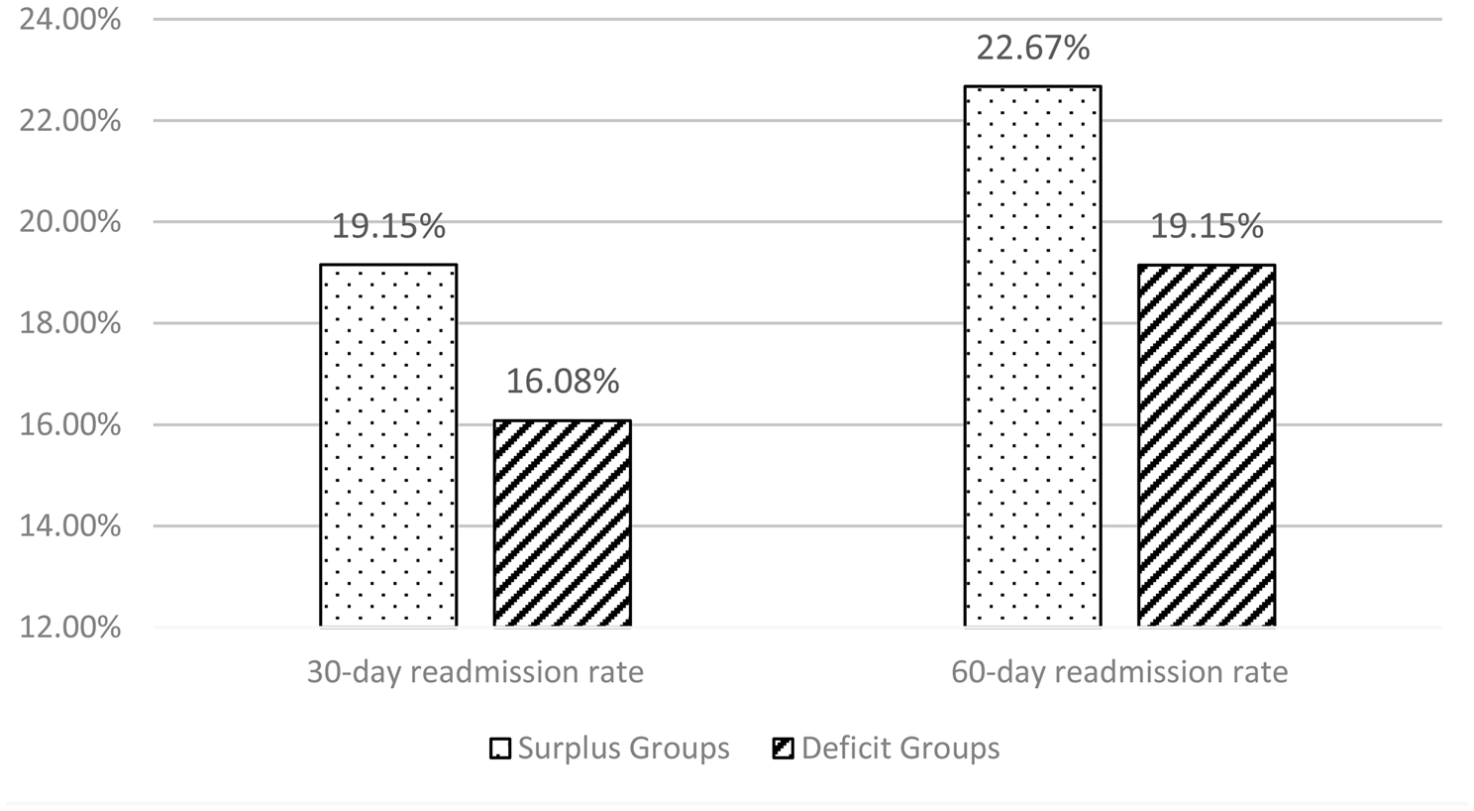
Comparison of healthcare quality between surplus and deficit groups. Data Source: Compiled by the author based on health insurance settlement sample data

Readmission rates were higher in the deficit group, indicating potential underprovision of services or lower quality care for patients in this group. Specifically, significant differences were observed between the deficit and surplus groups for 30-day and 60-day readmission rates (Fig. 3). Deficit group’s 30-day and 60-day readmission rates were 19.15% and 22.67%, respectively. And surplus group’s corresponding rates were 16.08% and 19.15%. The higher readmission rates in the deficit group may stem from stricter cost-control measures driven by financial pressures, which adversely affected treatment and follow-up care quality. Conversely, the surplus group benefitted from positive payment differentials, allowing providers to allocate additional resources and incentives to improve service quality and reduce readmission risks [5].

#### Breakpoint analysis: payment differentials and healthcare quality

**Fig. 4:**
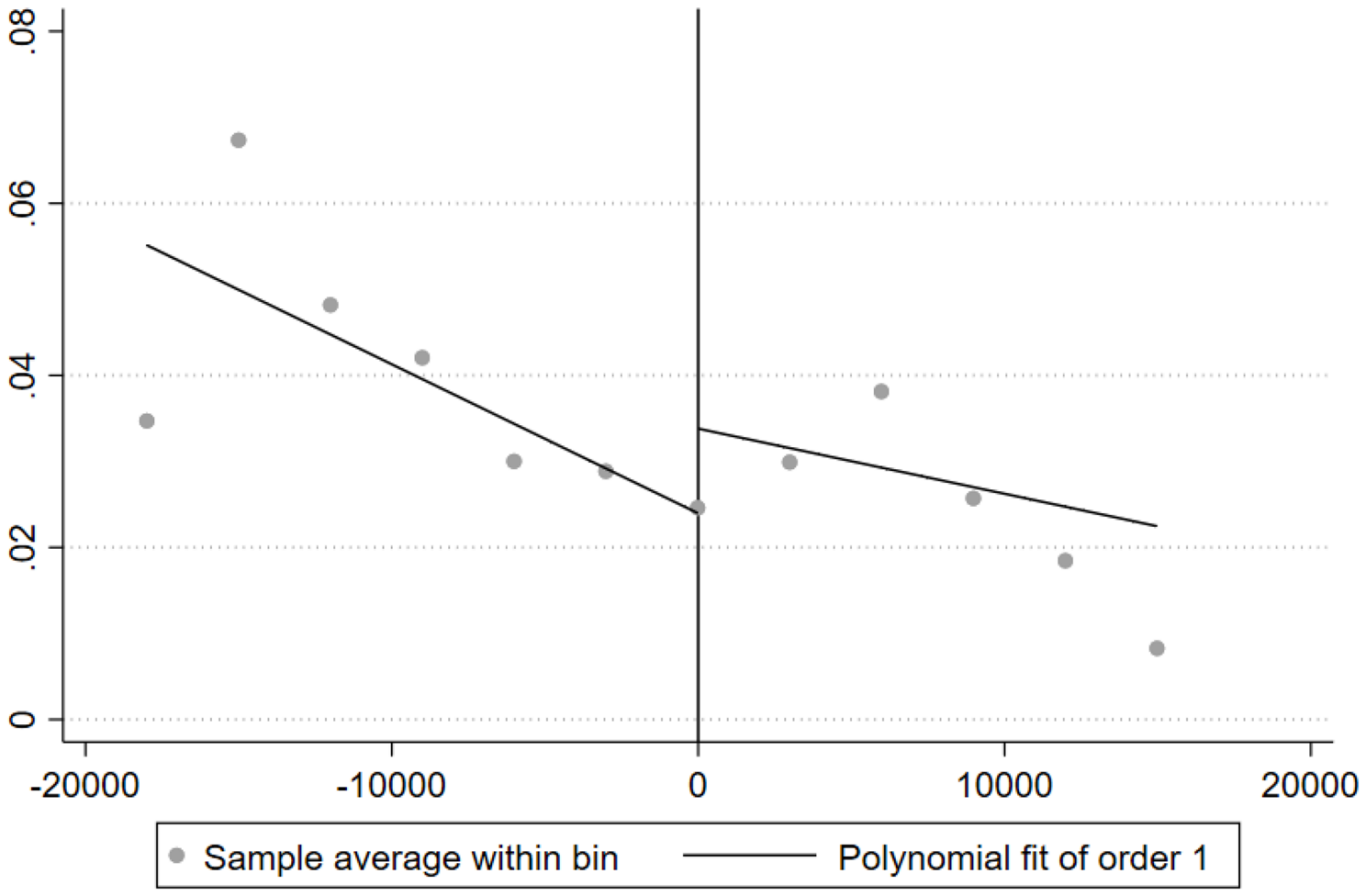
Breakpoint analysis of payment differentials and healthcare quality. Data Source: Compiled by the author based on health insurance settlement sample data

When the payment differentials is negative, providers may adopt extreme cost-saving measures, adversely affecting healthcare quality and leading to increased readmission rates. Conversely, positive payment differentials help enhance healthcare quality and lower readmission rates. As shown in Fig. 4, readmission rates gradually decrease with increasing payment differentials, reflecting improved healthcare quality. However, the slopes for the surplus and deficit groups differ significantly, indicating heterogeneity in how payment differentials affect readmission rates. When the payment differentials is negative, deficit group’s readmission rates increase sharply, as financial pressures may drive excessive resource compression and reduced treatment or care. This results in diminished healthcare quality and higher readmission rates.

#### Heterogeneity analysis of healthcare quality

**Fig. 5:**
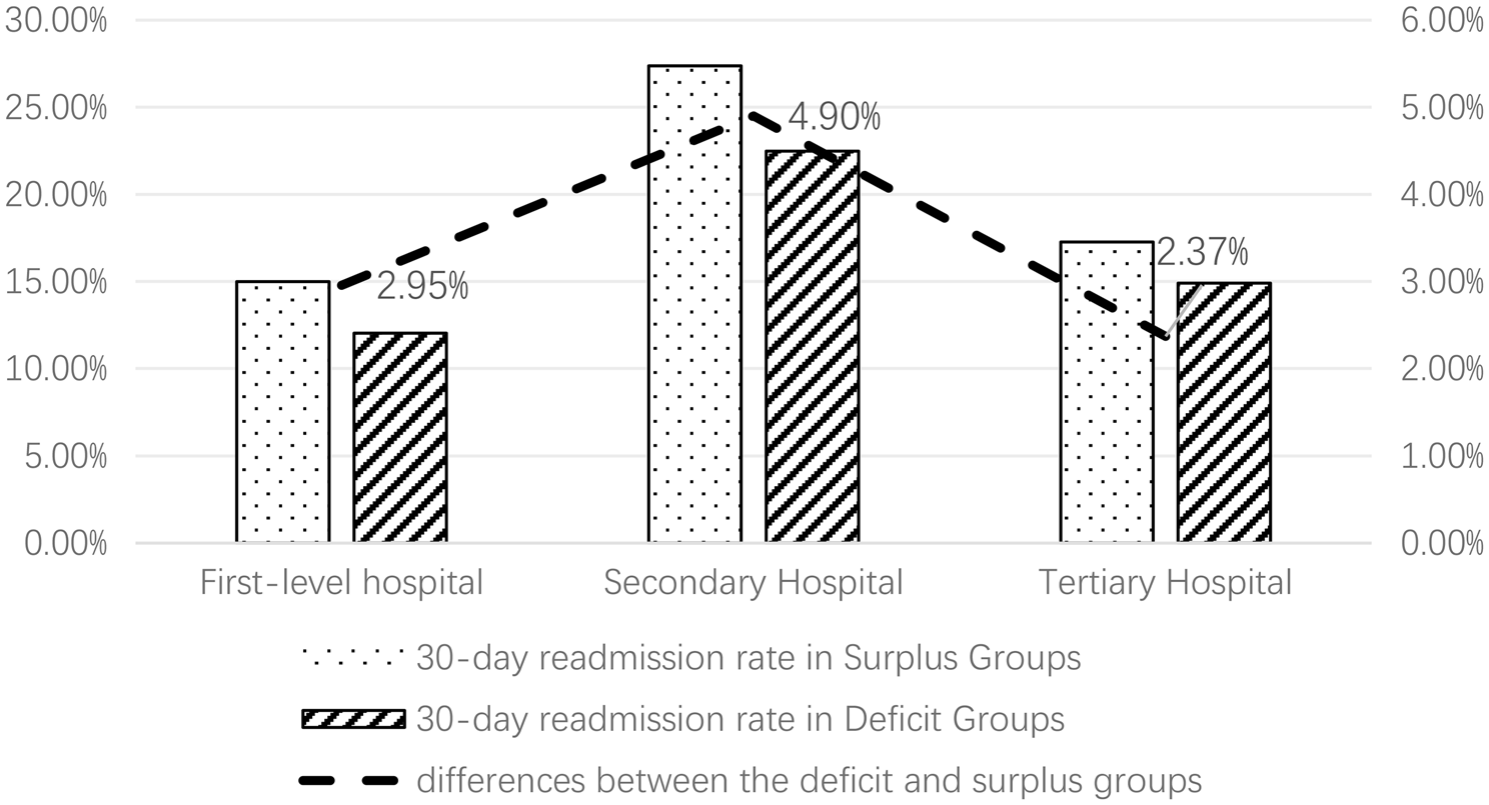
Healthcare quality comparison by hospital level. Data Source: Compiled by the author based on health insurance settlement sample data

Healthcare quality differences between the surplus and deficit groups vary significantly across hospital levels, treatment types, and patient characteristics.Readmission rates in secondary hospitals were generally higher than in primary and tertiary hospitals, with the largest gap of 4.9% points observed between the surplus and deficit groups (Fig. 5). Secondary Hospitals’ Patients in the surplus group experienced management challenges due to resource constraints, while those in the deficit group faced higher clinical risks from overuse of resources, increasing their readmission rates [34].

**Fig. 6:**
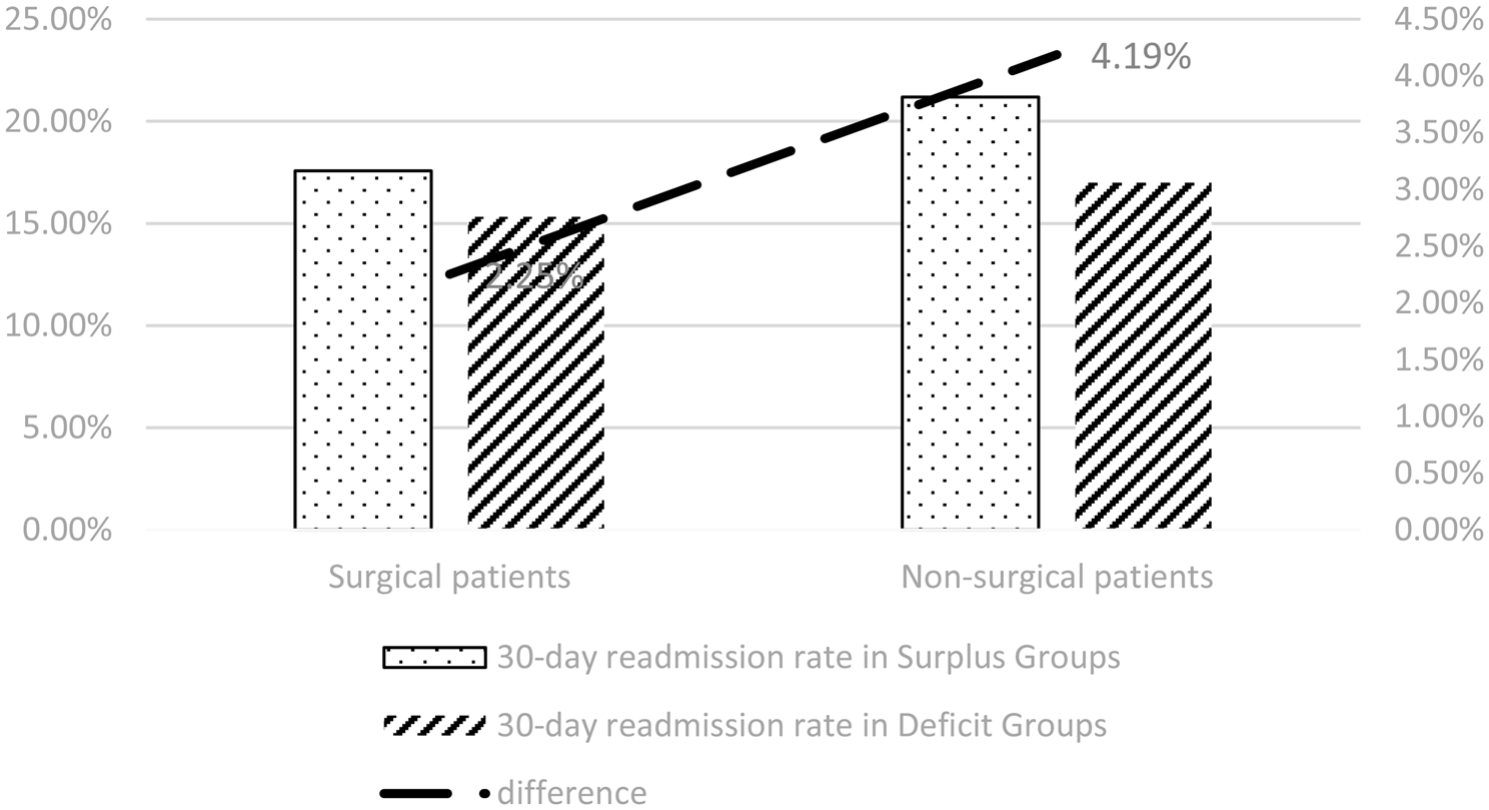
Healthcare quality comparison by treatment type. Data Source: Compiled by the author based on health insurance settlement sample data

Non-surgical patients exhibited higher readmission rates than surgical patients, with a 4.19% gap between the surplus and deficit groups (Fig. 6). Non-surgical patients often suffer from chronic or complex conditions with long treatment cycles and uncertain outcomes. In contrast, surgical patients undergo targeted interventions with clearer recovery pathways, which reduces uncertainty and leads to more predictable outcomes. This difference in treatment pathways exacerbates the disparities in utilization efficiency between the surplus and deficit groups, as surgical patients benefit more from the structured nature of surgical recovery, while non-surgical patients, whose conditions are often more unpredictable, face higher risks of complications and readmission [35].

**Fig. 7:**
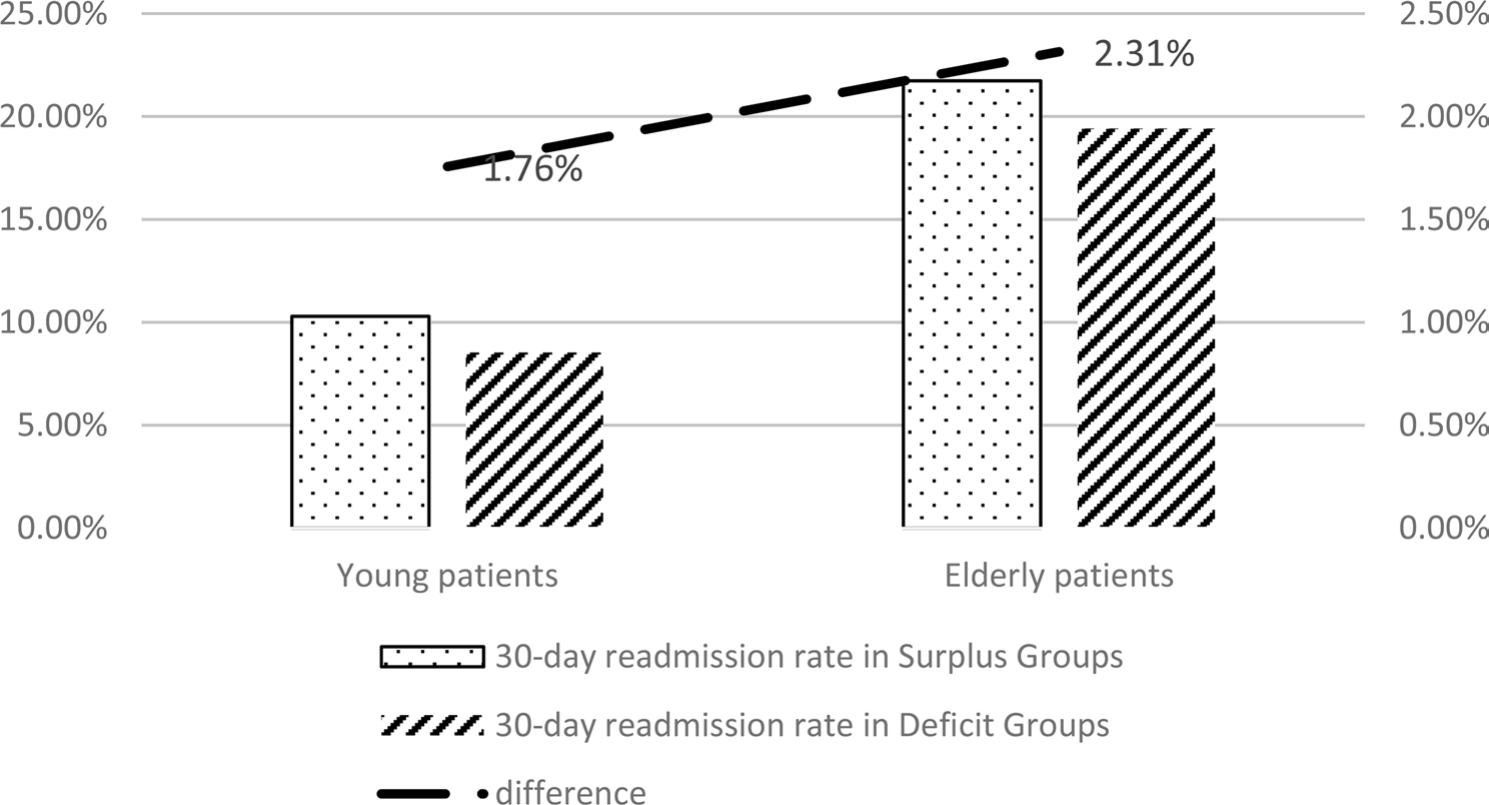
Healthcare quality comparison by patient age. Data Source: Compiled by the author based on health insurance settlement sample data

Elderly patients had significantly higher readmission rates than younger patients, with a 2.31% difference between the surplus and deficit groups (Fig. 7). Elderly patients typically present with multiple chronic conditions and declining physical function, which require more complex and comprehensive care. In the deficit group, excessive or unnecessary interventions may expose elderly patients to adverse effects or complications, further diminishing healthcare quality and increasing the likelihood of readmission [36].

These descriptive patterns provide preliminary insights and motivation for the empirical analysis.

### Baseline regression

Negative medical insurance payment differentials are significantly associated with higher readmission rates, providing empirical support for Hypothesis 1.Specifically, the regressions of Model 1 in Table 2 and Model 4 in Table 3 only include lnc, D1 and lnc*D1, and control the year effect and ADRG disease group effect. The results show that the coefficient of lnc is -0.6301 and the marginal coefficient is -0.072. It is significant at the 1% level, that is, for every 1% point increase in the medical insurance payment difference, the probability of patient readmission will decrease by 7.2% points; the coefficient of lnc*D1 is 1.9087, and the marginal coefficient is 0.218, which is significant at the 1% level. That is, for every percentage point reduction in the medical insurance payment difference, the probability of patient readmission will increase by 14.6 (-0.072 + 0.218 = 0.146) percentage points. Control variables such as gender and age were added to the regressions of Model 2 in Table 2 and Model 5 in Table 3. The results show that the coefficient of lnc is -0.2339 and the marginal coefficient is -0.025, which is significant at the 1% level, that is, every time the medical insurance payment difference increases by 1% point, the patient’s probability of readmission will be reduced by 2.5% points. The coefficient of lnc*D1 is 1.6104, and the marginal coefficient is 0.174, which is significant at the 1% level. That is, when the medical institution decreases by 1% point, the patient’s probability of readmission will be It will increase by 14.9 (-0.025 + 0.174 = 0.149) percentage points. When a medical institution has a surplus in medical insurance payments, the probability of a decrease in patient readmission rates is lower than the probability of an increase in the readmission rate when medical insurance payments are in deficit. This asymmetric pattern is consistent with the following possible explanations: Positive payment differentials (surplus) provide healthcare providers with additional resources, allowing them to optimize services, particularly in improving care quality and patient management. The financial cushion offered by surplus funds enables institutions to invest in high-quality treatments and effective follow-up care, which significantly reduces readmission rates. In contrast, negative payment differentials (deficit) create financial pressures that lead providers to adopt cost-cutting measures, such as reducing necessary diagnostic tests, shortening hospital stays, and limiting follow-up care. These cost-saving strategies can compromise treatment outcomes and recovery, leaving patients with unresolved health issues and increasing the likelihood of readmissions.

The findings suggest that financial incentives may play an important role in the design of DRG payment systems to maintain and improve healthcare quality.

**Table 2:** Regression analysis of medical insurance payment differentials on readmission rate

	Model 1	Model 2	Model 3
	30-day readmission rate	30-day readmission rate	30-day readmission rate
lnc	-0.6301***	-0.2339***	-0.0459
	(0.0500)	(0.0523)	(0.0527)
D1	-1.1528***	-0.9640***	-0.5625***
	(0.0778)	(0.0824)	(0.0875)
lnc*D1	1.9087***	1.6104***	0.8523***
	(0.1151)	(0.1232)	(0.1343)
log total medical expenses			-0.3805***
			(0.0230)
LOS			0.0570***
			(0.0013)
gender		0.1541***	0.1033***
		(0.0190)	(0.0195)
age		0.0278***	0.0240***
		(0.0009)	(0.0009)
hospital grade		-0.2761***	-0.1343***
		(0.0160)	(0.0183)
whether surgery		-0.1070***	-0.0218
		(0.0255)	(0.0265)
other diagnostic codes		0.1062***	0.1000***
		(0.0025)	(0.0026)
disease group fixed effect	fixed	fixed	fixed
year fixed effects	fixed	fixed	fixed
constant	-0.6648***	-3.9047***	-1.3199***
	(0.0951)	(0.1348)	(0.2604)
pseudo R2	0.2100	0.2543	0.2794
N	115,544	115,544	115,544

Note: *, **, and *** indicate significance at the 10%, 5%, and 1% significance levels respectively. The values ​​in parentheses are t values, the same as in the table below

^3^ It should be noted that the key explanatory variable is constructed as a ratio (in logarithmic form), and therefore small changes in the underlying ratio may correspond to relatively large marginal effects. These effects should be interpreted as relative changes rather than direct policy-relevant magnitudes

**Table 3:** Marginal regression analysis of medical insurance payment differentials on readmission rate

Margins	Model 4	Model 5	Model 6
	30-day readmission rate	30-day readmission rate	30-day readmission rate
lnc	-0.072***	-0.025***	-0.005
cd1	0.218***	0.174***	0.088***
N	115,544	115,544	115,544
pseudo R2	0.254	0.254	0.279

### Analysis of potential mechanisms

Healthcare providers primarily achieve surplus payments by adjusting the quantity and quality of services they offer, reflecting their behavioral tendencies under the health insurance payment system. Specifically, providers modify service content and scale to optimize surplus outcomes when faced with surpluses or deficits in payment differentials [37].

The intermediary variables logarithm of hospitalization days and total medical expenses were added to the regressions in Model 3 of Table 2 and Model 6 of Table 3. The lnc coefficient became small and insignificant, and the coefficients of hospitalization days and total medical expenses were significant, suggesting that these variables may be associated with both payment differentials and readmission outcomes. Table 4 presents the regression results of the second step of the causal-step regression approach (used here as an exploratory analysis) on the impact of the independent variables on the mediating variables, that is, the regression results on the impact of lnc, D1 and lnc*D1 on the logarithm of hospitalization days and total medical expenses. Controls were added to the model. variables, and controlled for year effects and ADRG disease group effects.

When medical insurance pays a surplus, the decline in medical expenses and hospitalization days is much lower than when it pays a deficit. Model 7 in Table 4 shows the regression results of lnc, D1 and lnc*D1 on the total logarithmic medical expenses. The coefficient of lnc is -0.5670, which is significant at the 1% level. That is, when the medical insurance payment differentials of medical institutions increases by 1% point, The total medical expenses of patients will be reduced by 0.5670%; the coefficient of lnc*D1 is -1.3666, which is significant at the 1% level. That is, when the medical insurance payment difference decreases by 1% point, the total medical expenses of patients will be reduced by 1.9336% (-0.5670-1.3666 = 1.9336). Model 8 in Table 4 shows the regression results of lnc, D1 and lnc*D1 on the number of days of hospitalization. The coefficient of lnc is 1.5620, which is significant at the 1% level. That is, when the medical institution makes a profit of 1% point, the number of days of hospitalization of the patient will increase by 1.562. Unit; the coefficient of lnc*D1 is 12.9144, which is significant at the 1% level. That is, when the medical insurance payment difference decreases by 1% point, the patient’s hospitalization days will increase by 14.4764 units (1.5620 + 12.9144 = 14.4764). In column (3) of Table 2, the impact of lnc, D1 and lnc*D1 on the readmission rate is significant at the 1% level, indicating a significant association between the variables. In column (1) of Table 4, lnc, D1 and lnc*D1 have a negative impact on the length of hospitalization and are significant at the 1% level, indicating that medical institutions reduce the quality of medical care by reducing total medical expenses. In column (2) of Table 4 lnc, D1 and lnc*D1 have a positive impact on hospitalization and are significant at the 1% level, indicating that medical institutions affect the quality of medical care by increasing the number of hospitalization days. Based on the above analysis, these results are consistent with potential pathways linking these variables between the length of hospitalization and total medical expenses and readmission rate.

The combined analysis reveals that LOS and total medical expenses serve as mediating variables in the relationship between the payment differentials and readmission rates. In Positive payment differentials (Surplus) Scenarios: Providers are more conservative in controlling medical expenses and LOS, optimizing resource allocation instead of aggressively reducing services or compressing LOS. Financial surpluses provide flexibility to improve efficiency and invest in quality enhancement. For example, optimized treatment plans and reasonable LOS management reduce unnecessary expenditures without compromising patient care quality. The slower reduction in expenses and LOS reflects a balance between quality assurance and cost control. In Negative payment differentials (Deficit) Scenarios: Providers face greater financial pressure and adopt more aggressive cost-control measures, such as shortening LOS, reducing diagnostic tests, and compressing follow-up care. These actions significantly reduce medical expenses but may compromise treatment efficacy and recovery processes. For instance, restrictive treatments aimed at short-term cost savings may exacerbate health risks, leading to dramatic increases in LOS due to complications. Financial constraints shift the focus toward cost reduction rather than quality assurance, potentially sacrificing treatment outcomes. Healthcare institutions exhibit asymmetric responses to payment differentials, with surplus scenarios enabling gradual quality improvement, while deficit scenarios prompt aggressive cost-cutting that adversely impacts healthcare quality. These findings highlight the critical need for balanced payment mechanisms to ensure both financial sustainability and optimal patient outcomes.

**Table 4:** Regression analysis of medical insurance payment differentials on total medical expenses and hospitalization days

	Model 7	Model 8
	log total medical expenses	LOS
lnc	-0.5670***	1.5620***
	(0.0080)	(0.1023)
D1	0.8433***	-6.9829***
	(0.0123)	(0.1935)
lnc*D1	-1.3666***	12.9144***
	(0.0203)	(0.3168)
gender	-0.0142***	0.5704***
	(0.0024)	(0.0429)
age	0.0007***	0.0366***
	(0.0001)	(0.0016)
hospital grade	0.3099***	-3.5144***
	(0.0023)	(0.0466)
whether surgery	0.0968***	-0.9867***
	(0.0031)	(0.0588)
other diagnostic codes	0.0232***	0.0021
	(0.0004)	(0.0069)
disease group fixed effect	fixed	fixed
year fixed effects	fixed	fixed
constant	9.5439***	-80.2266***
	(0.0286)	(0.9845)
R2	0.7777	0.6445
F	4691.6594	1508.1434
N	115,544	115,544

This article further uses the Bootstrap method as an additional exploratory analysis of potential mechanisms. The results are shown in Table 5. It mainly relies on observing whether the indirect effect contains 0 within the 95% confidence interval. If the test result contains 0, the indirect effect is not significant, that is, there is no mediating effect, and the number of samples in Bootstrap is set to 1000. Bootstrap test showed that it had a significant impact on readmission rate through length of stay and total medical expenses. The confidence intervals of all two paths do not include 0, indicating that the indirect effect exists significantly. The effect sizes of hospitalization days and total medical expenses are − 0.05684859 and − 0.0069485 respectively.

**Table 5:** Bootstrap analysis

Mediator	Effect	95% BootLLCI	95% BootULCI	Test Results
LOS	− 0.05684859	− 0.0590876	− 0.0542524	establishment
log total medical expenses	− 0.0069485	− 0.0107153	− 0.0031817	establishment

### Robustness check

This article adjusts the core independent variables and replaces the core independent variables with r_c value and r_c value squared. The coefficient of r_c in model 9 in Table 6 is -0.0115, the coefficient of r_c squared is -0.0597, and the quadratic term coefficient is significant, proving that there is an inflection point of -2.56., indicating that health insurance payment balances affect readmission rates. This article adjusts the core dependent variable and replaces the core independent variable with the 60-day readmission rate. The results of Model 10 in Table 6 and Model 12 in Table 7 show that the coefficient of lnc is -0.0978 and the marginal coefficient is -0.012, which is significant at the 1% level. It means that for every 1% point increase in the medical insurance payment difference, the probability of patient readmission in 60 days will decrease by 1.2% points. The coefficient of lnc*D1 is 1.4413, and the marginal coefficient is 0.172, which is significant at the 1% level, that is, the medical insurance payment When the difference decreases by 1% point, the patient’s probability of 60-day readmission will increase by 0.06 (-0.012 + 0.172 = 0.06) percentage points.

**Table 6:** Regression analysis of the change ratio of medical institution income on readmission rate

	Model 9	Model 10
	30-day readmission rate	30-day readmission rate
r_c	-0.0115	
	(0.0225)	
r_c2	-0.0597***	
	(0.0111)	
lnc		-0.0978**
		(0.0488)
D1		-0.8192***
		(0.0779)
cd1		1.4413***
		(0.1171)
lnc	0.1535***	0.0949***
	(0.0190)	(0.0180)
D1	0.0282***	0.0279***
	(0.0009)	(0.0008)
lnc*D1	-0.2950***	-0.3320***
	(0.0159)	(0.0152)
gender	-0.1142***	-0.0715***
	(0.0255)	(0.0239)
age	0.1055***	0.0977***
	(0.0025)	(0.0024)
hospital grade	fixed	fixed
	fixed	fixed
whether surgery	-4.0187***	-3.6334***
	(0.1245)	(0.1283)
pseudo R2	0.2532	0.2596
N	115,544	115,544

**Table 7:** Marginal regression analysis of the change ratio of medical institution income on readmission rate

	Model 11	Model 12
	30-day readmission rate	30-day readmission rate
r_c	-0.001	
lnc		-0.012**
cd1		0.172***
N	115,544	115,544
pseudo R2	0.253	0.260

### Robustness check

#### Hospital grade

The effect of the payment differentials on healthcare quality varies across different hospital levels, closely linked to each institution’s positioning and service characteristics. Primary and secondary hospitals are more susceptible to fluctuations in payment differentials, while tertiary hospitals tend to remain relatively stable. As shown in Table 8, for first-level medical institutions, the coefficient of lnc is -2.0688, which is significant at the 1% level. That is, when the medical insurance payment difference increases by 1% point, the probability of patient readmission will decrease, lnc* The coefficient of D1 is 1.0515, which is significant at the 1% level. That is, when the medical insurance payment difference decreases by 1% point, the probability of patient readmission will decrease. For secondary medical institutions, the coefficient of lnc is -1.279, which is significant at the 1% level. That is, when the medical insurance payment difference increases by 1% point, the probability of patient readmission will decrease. The coefficient of lnc*D1 is 2.8965. It is significant at the 1% level, that is, every time the medical insurance payment differentials decreases by 1% point, the probability of patient readmission will increase. For tertiary medical institutions, the impact of payment difference on medical quality is not significant. The main reason for this difference could be that tertiary hospitals generally exhibit superior efficiency in healthcare service delivery and quality management. They tend to achieve higher fund surpluses through optimizing medical processes and resource allocation, meaning that payment differentials fluctuations have a weaker impact on the quality of services. In contrast, primary and secondary hospitals are more likely to cut healthcare resources to balance budgets, significantly affecting service quality and increasing readmission rates.

**Table 8:** Regression analysis of medical insurance payment difference and readmission rate by hospital grade

30-day readmission rate	Model 13	Model 14	Model 15
	First-level medical institution	Secondary medical institutions	Tertiary medical institutions
lnc	-2.0688***	-1.2790***	-0.0704
	(0.3907)	(0.1798)	(0.0551)
D1	-1.0897***	-1.9748***	-0.8110***
	(0.4179)	(0.2168)	(0.0935)
lnc*D1	1.0515*	2.8965***	1.4412***
	(0.5676)	(0.3136)	(0.1413)
control variables	control	control	control
disease group/year fixed effects	fixed	fixed	fixed
constant	-3.2118***	-6.2041***	-3.9490***
	(1.2098)	(0.3918)	(0.1425)
pseudo R2	0.1725	0.2727	0.2732
N	7807	21,972	85,344

#### Age

The effect of the payment differentials varies significantly across different age groups. As shown in Table 9, for young patients, the coefficient of lnc is -0.5010, which is significant at the 1% level. That is, when the medical insurance payment difference increases by 1% point, the probability of patient readmission will decrease. The coefficient of lnc*D1 The coefficient is 2.1062, which is significant at the 1% level. That is, when the medical insurance payment difference decreases by 1% point, the probability of patient readmission will increase. For elderly patients, readmission rates are often more influenced by chronic diseases or complex conditions, with a smaller effect from payment differentials fluctuations. Therefore, the payment differentials does not significantly impact the healthcare quality for older patients. This suggests that while the payment differentials influences the treatment and management of younger patients, elderly patients’ care needs are primarily driven by medical complexity, which diminishes the direct effect of payment differentials on their outcomes.

**Table 9:** Regression analysis of the difference in medical insurance payment by age and the rate of readmission

30-day readmission rate	Model 16	Model 17
	Young patients	Elderly patients
lnc	-0.5010***	0.0622
	(0.0627)	(0.0968)
D1	-1.3059***	-0.4992***
	(0.0920)	(0.1888)
cd1	2.1062***	0.7380***
	(0.1360)	(0.2857)
control variables	control	control
disease group/year fixed effects	fixed	fixed
constant	-1.5491***	-1.6637***
	(0.1211)	(0.4834)
pseudo R2	0.1986	0.3995
N	84,236	31,098

#### Surgical treatment

The effect of the payment differentials on healthcare quality also varies significantly based on whether patients undergo surgery, with non-surgical patients being more strongly affected. As shown in Table 10, for patients who have not undergone surgery, the coefficient of lnc is -1.2985, which is significant at the 1% level. That is, when the medical insurance payment difference increases by 1% point, the probability of patient readmission will decrease, lnc * The coefficient of D1 is 2.5213, which is significant at the 1% level. That is, when the medical insurance payment difference decreases by 1% point, the probability of patient readmission will increase. For patients undergoing surgery, the coefficient of lnc is 0.1084, which is significant at the 10% level. That is, when the medical insurance payment difference increases by 1% point, the probability of patient readmission will increase. The coefficient of lnc*D1 is 1.3747, at It is significant at the 1% level, that is, when the medical insurance payment difference decreases by 1% point, the probability of patient readmission will increase. For non-surgical patients, the payment differentials and readmission probability are negatively correlated, possibly because healthcare providers focus on improving service quality and reducing readmission rates, which consequently lowers the risk of readmission. However, for surgical patients, the relationship is positive: a surplus in the payment differentials is associated with an increase in readmission rates. This could be due to the higher complexity of surgical patients, where providers, aiming to improve treatment quality, may intervene more aggressively, leading to an increased readmission probability when surplus funds are available.

**Table 10:** Regression analysis of medical insurance payment difference and readmission rate by treatment method

30-day readmission rate	Model 18	Model 19
	No surgery	surgery
lnc	-1.2985***	0.1084*
	(0.1054)	(0.0642)
D1	-1.7499***	-0.7280***
	(0.1400)	(0.1042)
cd1	2.5213***	1.3747***
	(0.2037)	(0.1554)
control variables	control	control
disease group/year fixed effects	fixed	fixed
constant	-0.5907	-3.2962***
	(0.3778)	(0.1873)
pseudo R2	0.2011	0.2944
N	51,147	64,206

## Discussion

The DRG payment system, through differentiated payment standards and surplus retention mechanisms, has contributed to curbing over-medicalization and enhancing the transparency and efficiency of healthcare services. However, the system still encounters notable challenges. In certain situations, the effectiveness of cost control is limited, and insufficient incentives may drive healthcare institutions to engage in undesirable behaviors such as upcoding, “cherry-picking,” reducing necessary services, or shortening hospital stays. These responses may compromise treatment quality and negatively affect patient health outcomes. Meanwhile, the emphasis on cost containment often exceeds the motivation to improve service quality, making it difficult to maintain a balance between efficiency and quality.

The empirical findings of this study underscore the significant role of payment differentials in shaping healthcare quality. When payment differentials are negative, readmission rates rise substantially, indicating that inadequate reimbursement encourages hospitals to adopt more aggressive cost-control measures. These measures manifest through decreased expenditures and shortened lengths of stay, ultimately weakening the quality of care. In contrast, positive payment differentials provide institutions with greater financial capacity to optimize service delivery and uphold quality standards. Furthermore, the relationship between payment differentials and readmission outcomes differs across surgery types, hospital levels, and patient age groups, demonstrating substantial heterogeneity in institutional responses. These results highlight the importance of refining reimbursement design to prevent quality deterioration, particularly among resource-constrained institutions or vulnerable patient groups.

## Conclusion

This study shows that payment differentials within the DRG payment system are significantly associated with healthcare quality outcomes. When the payment differential is negative, patient readmission rates are significantly higher. This pattern is consistent with changes in medical behaviors, including lower medical expenditures and shorter lengths of stay. These findings suggest that insufficient reimbursement may be associated with more aggressive cost-control strategies, which may in turn be associated with changes in quality of care. At the same time, when the payment differential is positive, healthcare institutions may have greater financial flexibility, which may be associated with maintaining service quality and improving treatment processes. The impact of payment differentials also varies across surgery types, hospital levels, and patient age groups, suggesting heterogeneous responses associated with the incentive structure.

Based on these findings, several policy implications may be considered. First, improving the alignment between medical service prices and health insurance payment standards may be important. DRG/DIP payment standards that rely primarily on historical average costs often fail to reflect actual treatment costs, differences in patient characteristics, or variations in resource allocation across institutions. A more scientific and dynamic approach to setting payment standards may be needed to help maintain payment differentials within a reasonable range. Second, healthcare institutions may benefit from optimizing treatment pathways and improving resource efficiency to maintain service quality while controlling costs. Strengthening disease management, reducing unnecessary procedures, and refining service processes may help institutions achieve reasonable cost control without compromising quality. Third, it may be beneficial to promote a shift toward a value-based payment system. Linking payment more closely to patient outcomes, service effectiveness, and quality indicators may help reduce the risk that hospitals reduce necessary services in the pursuit of cost savings. A value-oriented mechanism may help reduce the sensitivity of readmission rates to payment differentials and may contribute to a more balanced relationship between cost control and service quality.

### Limitations

This study has certain limitations. Firstly, The payment differential is measured as a log-transformed ratio, which may not be directly intuitive for policy interpretation. Due to data limitations, more interpretable measures based on monetary surplus or standardized costs are not available.Secondly, Due to data availability constraints, only readmission rates were used as a proxy for healthcare quality. While readmission rates reflect healthcare effectiveness to some extent, they are a narrow metric and may not fully capture the multidimensional aspects of healthcare quality, especially with respect to patient satisfaction, rehabilitation outcomes, and long-term health impacts. Future research should expand the range of indicators used to assess healthcare quality and explore more comprehensive metrics to better capture the broader dimensions of healthcare service performance. Meanwhile, although we control for extensive observable characteristics including patient demographics, clinical features, hospital characteristics, and disease group and year fixed effects, we are unable to obtain more detailed clinical information (such as Elixhauser conditions and other healthcare conditions) due to data limitations. These unobserved patient health conditions and treatment characteristics may simultaneously affect both payment differentials and readmission rates, resulting in residual confounding. Thirdly, since the payment differential may also be influenced by treatment decisions and patient characteristics, it cannot be interpreted as a purely exogenous measure of incentives. Fourthly, It should be noted that total medical expenses may not fully reflect true economic costs and may be influenced by provider behavior and patient characteristics. As a result, the payment differential in this study should be interpreted as the gap between reimbursement and observed expenditure, rather than a precise measure of profit or cost. This limitation should be considered when interpreting the empirical results.

## Data Availability

The datasets used in this study are from the health insurance settlement system of DRG pilot cities. Due to data privacy and confidentiality agreements with the provider, the datasets are not publicly available but can be obtained from the corresponding author on reasonable request (with permission from the data provider).

## Footnotes

1 Total medical expenses refer to the service-based charges recorded in administrative claims data under the fee-for-service (FFS) model, which reflect actual resource use and treatment costs incurred by medical institutions and serve as the benchmark for comparison with DRG payments to calculate payment differentials.

2 This specification follows Du (2017) [25], who adopts a multiplicative structure to capture the interaction between health and economic variables in the context of health insurance incentives.The multiplicative form reflects the complementarity between health and income: the marginal utility of income depends on health status, and vice versa. When health is poor, the utility derived from income is limited, whereas better health enhances the ability to generate and utilize income. This assumption is supported by the state-dependent utility literature, which shows that the marginal utility of income varies with health conditions [26]. Empirical evidence further suggests positive cross-partial effects between health and consumption, indicating complementarity in preferences [27]. It is also consistent with the health capital framework [28, 29], where health affects productivity and income generation, while income influences health investment, implying a non-separable relationship between the two [30, 31].

## Abbreviations

DRG: Diagnosis Related Groups
